# Primary Appendiceal Adenocarcinoma Presenting with Hematochezia due to the Invading Tumor in the Sigmoid Colon

**DOI:** 10.1155/2020/8833573

**Published:** 2020-09-07

**Authors:** Tatsuya Suzuki, Yasuhiro Yamamoto, Toshihiko Torigoe, Shoichiro Mizukami, Kengo Shigehara

**Affiliations:** ^1^Department of Surgery, Kobayashi Hospital, 4-2, Kita-3-Jonishi, Kitami, Hokkaido 090-8567, Japan; ^2^Department of Pathology, Sapporo Medical University School of Medicine, South-1, West-17, Chuo-ku, Sapporo, Hokkaido 060-8556, Japan

## Abstract

Primary appendiceal tumors are rare malignancies; some cases have been described to invade other organs, and this represents a very rare clinical condition. We report a case of appendiceal adenocarcinoma invading the sigmoid colon and a review of similar cases. A 69-year-old woman with complaints of hematochezia was admitted to the hospital. Colonoscopy revealed a tumor in the sigmoid colon, which was a well-differentiated tubular adenocarcinoma. A computed tomography scan showed an appendiceal mass that involved the sigmoid colon, suggesting an appendiceal cancer invading the sigmoid colon. Ileocecal resection with extended lymphadenectomy and en bloc resection of the sigmoid colon was performed. The appendiceal tumor involved the sigmoid colon and the terminal ileum. The ileocecal part which included the tumor and the involved sigmoid colon was resected in total. Macroscopic findings showed that the appendiceal tumor made a fistula with the sigmoid colon. Pathological examination revealed that the tumor was a well-differentiated tubular adenocarcinoma that invaded the sigmoid colon. The final pathological stage was T4bN0M0, stage IIC. The patient was discharged from the hospital uneventfully. She was alive without relapse after a 20-month follow-up. Although an appendiceal tumor invading the rectosigmoid region is rare, a preoperative diagnosis can be obtained that facilitates the planning of a suitable surgical procedure: en bloc resection of the ileocecal part and the rectosigmoid part.

## 1. Introduction

Primary appendiceal tumors are rare malignancies with an age-adjusted incidence of 4 per 1,000,000 people per year, and these tumors account for 0.4 to 1 percent of all gastrointestinal malignancies [1, 2]. Some cases of appendiceal cancer have been described as invading or making a fistula with other organs, which represent a very rare clinical condition. We report a case of appendiceal adenocarcinoma invading the sigmoid colon presenting with hematochezia and a review of the literature concerning similar cases.

## 2. Case Presentation

**Figure 1:**
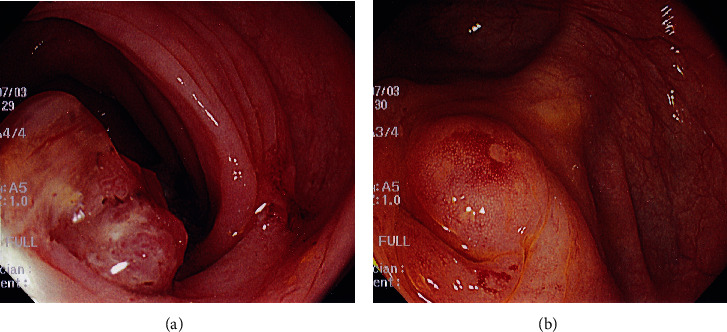
Colonoscopic findings. (a) A polypoid-type tumor was seen in the sigmoid colon, which was initially assumed to be a sigmoid colon cancer. (b) An elevated lesion with redness was observed in the appendiceal orifice, where biopsies were not performed.

**Figure 2:**
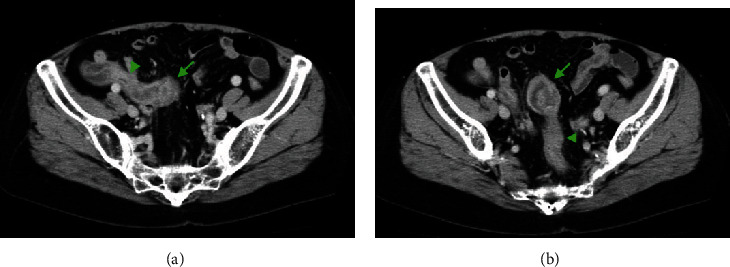
CT findings. (a) An appendiceal mass was seen at the distal part of the appendix. The appendiceal mass and the appendix are indicated by an arrow and an arrowhead, respectively. (b) The appendiceal mass involved the sigmoid colon. The mass and the normal rectosigmoid region are indicated by an arrow and an arrowhead, respectively.

A 69-year-old woman with a chief complaint of hematochezia and anorexia that had been present for one month was admitted to the hospital. Physical examination and blood test results revealed nothing of note except for an elevated carcinoembryonic antigen of 24.7 ng/mL. Colonoscopy revealed that a type 1 tumor, i.e., a polypoid-type tumor, was located in the sigmoid colon, and an elevated lesion with redness was located in the appendiceal orifice (Figure 1). The tumor was biopsied and diagnosed with well-differentiated tubular adenocarcinoma, which was suspicious of primary sigmoid colon cancer. The elevated lesion in the appendiceal orifice had redness on its top surface, but biopsy was not performed there because it was not suspected to be malignant due to the apparently normal mucosa. A computed tomography (CT) scan showed, however, an appendiceal mass that involved the sigmoid colon (Figure 2) and was located adjacent to the right ureter, suggesting an appendiceal cancer invading the sigmoid colon. The preoperative diagnosis was appendiceal cancer with a clinical stage of T4bN0M0, stage IIC, according to the TNM classification, the Union for International Cancer Control (UICC) 8th edition.

Ileocecal resection with extended lymphadenectomy and en bloc resection of the sigmoid colon was performed through a lower abdominal midline incision after the insertion of a ureteral stent. The appendiceal tumor involved the sigmoid colon and the terminal ileum. The ileocecal part was mobilized from the retroperitoneum, keeping the right ureter intact. The ileum was divided proximal to the involved part with a linear stapler. The ileocolic vessels were ligated at their origins, and the extended D3 lymph node dissection was performed. The ascending colon and then the sigmoid colon proximal to the invaded part were divided with a linear stapler. The ileocecal part which included the tumor and the involved sigmoid colon was resected in total after the rectum was divided. The pericolic lymph nodes were dissected in the resected area of the sigmoid mesocolon. The sigmoid colon and the rectum were anastomosed in a side-to-end fashion with a circular stapler, and the ileum and the ascending colon were anastomosed in a functional end-to-end fashion. The incision was closed after the placement of Blake™ silicone drains in the Douglas pouch and in the right lateral abdomen.

**Figure 3:**
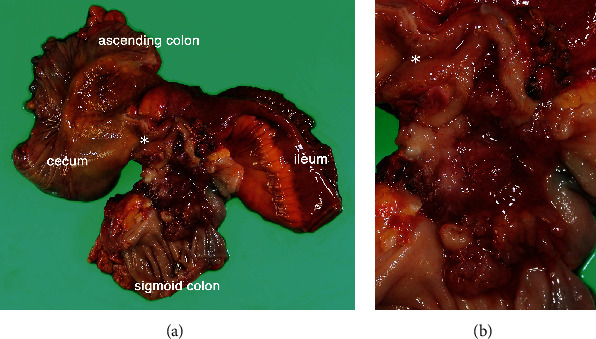
Macroscopic views of the resected specimen. (a) A tumor of 60 × 40 mm in size was seen in the distal part of the appendiceal lumen and extended into the lumen of the sigmoid colon with fistulation. The root of the appendix is indicated by an asterisk. (b) A closer view.

**Figure 4:**
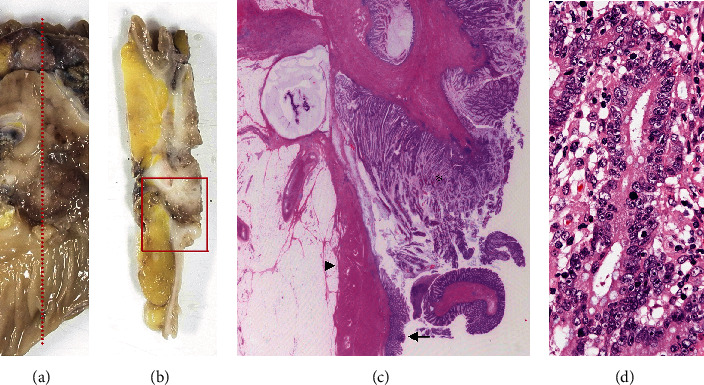
Microscopic findings of the resected specimen. (a) The specimen was cut along a red dotted line which included the appendiceal tumor and the invaded sigmoid colon. (b) A cross-section of the cut specimen. The area surrounded by a red box was examined microscopically. (c) A microscopic view. The tumor (indicated by an asterisk) had come into contact with the inner side of the sigmoid colon. The muscularis propria and the mucosa of the sigmoid colon are indicated by an arrowhead and an arrow, respectively. (d) A higher magnification view showing that the tumor was a well-differentiated tubular adenocarcinoma.

Macroscopic findings showed that the tumor measured 60 × 40 mm and invaded and made a fistula with the sigmoid colon (Figure 3). Pathological examination revealed that the tumor was a well-differentiated tubular adenocarcinoma according to the Japanese classification [3] and invaded the sigmoid colon (Figure 4). The tumor invaded in the broad area of the appendiceal mucosa and the largest part of the tumor existed in the appendix, whereas only a small part of the tumor was exposed in the mucosa of the sigmoid colon. In addition, the tumor appeared to invade from the peritoneal side toward the sigmoid colon lumen. These findings showed that the tumor originated from the appendix and did not originate from the sigmoid colon. All 41 resected lymph nodes, including lymph node No. 201, 202, and 203, were devoid of metastasis [3]. The pericolic lymph nodes in the sigmoid mesocolon (No. 241) were not explored for pathological examination. The final pathological stage was T4bN0M0, stage IIC, according to the TNM classification, UICC 8th edition.

The patient was discharged from the hospital uneventfully. Adjuvant chemotherapy was not performed, and she was alive without cancer relapse after a 20-month follow-up.

## 3. Discussion

We experienced a rare case of primary appendiceal adenocarcinoma that invaded and made a fistula with the sigmoid colon and was surgically treated successfully. Sixteen cases of appendiceal tumors invading the sigmoid colon or rectum were reviewed from the literature. Of these cases, 7 reports written in English were identified by a PubMed search using keywords such as “appendiceal cancer,” “appendiceal carcinoma,” “appendiceal adenocarcinoma,” “sigmoid colon,” “rectum,” “invasion,” “invading,” or “fistula” as well as using a manual search of references from the publications, and 9 reports written in Japanese were identified by Ichushi-Web search using keywords such as “appendiceal cancer,” “sigmoid colon,” or “rectum” (Table 1) [4–19]. A fistula was formed between the appendix and the colorectum in 12 cases, including our case, and there was no description of the fistulas in the other five cases. The histological type was described according to the World Health Organization (WHO) Classification of Tumours [20]. The term “cystadenoma” was replaced with the term “low-grade appendiceal mucinous neoplasm.” Additionally, the term “cystadenocarcinoma” was replaced with “appendiceal mucinous neoplasm (AMN),” regarding a tumor with pushing invasion, or “mucinous adenocarcinoma (MA),” regarding a tumor with infiltrative invasion [21]; the type of invasion was not mentioned in several reports, and in these cases, the type was described as “AMN or MA.” The term “well differentiated adenocarcinoma” or “well differentiated tubular adenocarcinoma” described in the Japanese classification was replaced with the term “adenocarcinoma not otherwise specified” [3, 22].

Of the 17 cases listed in Table 1, 10 cases had hematochezia, and one case had a positive fecal occult blood test. On the other hand, the most common presentation in primary appendiceal tumors was the right lower quadrant pain, which was often diagnosed as acute appendicitis. Nitecki et al. reported 94 noncarcinoid adenocarcinoma of the appendix; their presentation included acute right lower quadrant pain in 47 patients, a palpable mass in 13, ascites in 10, nonspecific gastrointestinal, or genitourinary complaints in five, and the other 19 patients were incidentally diagnosed at the time of surgery for an unrelated medical condition [23]. In addition, Ito et al. reported 36 patients of appendiceal adenocarcinoma; 25 patients presented with right lower quadrant pain, seven had palpable mass, four had nausea and vomiting, three had pelvic discomfort, one had weight loss, and one was diagnosed incidentally during other surgery [24]. These case series reported no patients who presented with hematochezia. Therefore, hematochezia seems to be a peculiar manifestation of appendiceal tumors when invading or making a fistula with the sigmoid colon or rectum.

A preoperative diagnosis of appendiceal tumors is difficult to make [23, 24]. This is probably because the appendiceal tumor represents a rare condition and is often mistaken for acute appendicitis [25], and these tumors are rarely detected by colonoscopy [26]. However, when an appendiceal tumor invaded and made a fistula with the colorectum, colonoscopy detected a polyp, an elevated lesion or a mass in the involved part (Table 1). Such colonoscopic findings, with or without a histologic diagnosis of malignancy, led to a preoperative diagnosis of appendiceal or colorectal cancer when combined with a CT scan. CT scans were performed in 13 cases; an appendiceal mass or dilatation, an ileocecal mass, or a pelvic mass was observed in 12 cases. Seven cases were preoperatively diagnosed as appendiceal cancer from the findings of appendiceal mass or dilation in 6 cases, whereas 4 cases were not diagnosed preoperatively as appendiceal cancer possibly due to failing to locate the exact site of a pelvic mass. Therefore, the findings of a mass or dilation in the appendix were helpful for the preoperative diagnosis of appendiceal tumor. Hence, although appendiceal tumors invading the sigmoid colon or rectum represents a rare clinical condition, the findings from colonoscopy and CT scans can lead to a proper diagnosis prior to treatment with this unusual condition in mind.

Regarding the surgical procedure, en bloc resection of both the ileocecal part and the rectosigmoid part is warranted for curative treatment. The extent of resection for appendiceal tumor differs among histological types [21]. In the case of low-grade appendiceal mucinous neoplasm (LAMN), which is classified as a tumor of borderline behavior in the WHO Classification of Tumours and was formerly classified as mucinous cystadenocarcinoma or mucinous cystadenoma [20], simple appendectomy is considered sufficient for LAMNs that are confined to the appendix, and a positive margin at the base of the appendix may require additional cecal resection [21]. In contrast, appendiceal adenocarcinomas, such as mucinous adenocarcinoma and adenocarcinoma not otherwise specified, warrant treatment by right hemicolectomy with lymph node dissection [21, 23]. Although systematic lymphadenectomy in the invaded rectosigmoid region was not documented nor performed in all 17 cases in Table 1, the necessity of extended lymph node dissection in the invaded region is unclear. In the present case, the lack of lymph node swelling in the ileocolic region preoperatively led us to conjecture that the possibility of lymphatic spread in the sigmoid region was minimal. In the case reported by Kumon et al., meanwhile, the tumor had metastasis in the pericolic lymph node of the invaded rectum (No. 251) [11]. Systematic lymphadenectomy might be necessary in such cases if the metastasis occurred via the lymphatic system in the invaded region, as discussed by Toyozumi et al. [27]. Additionally, in the case reported by Tokai et al., the tumor recurred in the paraaorta lymph node 18 months after surgery; the authors surmised that the extensive lymphadenectomy in the invaded region might have prevented the disease recurrence [19]. Evaluation of the lymph nodes in the invaded region and the longer follow-up of patients are needed to investigate this problem. Laparoscopic-assisted sigmoidectomy and en bloc right hemicolectomy have been reported by Stojanovic et al. [13], and laparoscopic ileocecal resection for appendiceal cancer with an ileal fistula has also been reported by Mukohyama et al. [28], suggesting that laparoscopic procedures can be treatment options for appendiceal tumors invading other organs. In any case, we believe that a proper diagnosis allows us to plan an appropriate surgical procedure in advance.

Of the 17 cases listed, peritoneal spread was observed in three cases, two of which had a poor prognosis. On the other hand, neither lymphatic nor peritoneal metastasis was seen in nine cases. This may be explained by the borderline behavior of LAMNs or by the prevention of free intraperitoneal spillage of neoplastic cells through the formation of a fistula to another organ [17]. In addition, the symptoms associated with blood discharge caused by the fistula may lead to further investigation and early detection of resectable tumors [29].

This case report and literature review are limited because the cases searched are only from case reports and short case series within a few decades, and other cases possibly contained in a larger case series or in older literature may have been missed. Nonetheless, the case of an appendiceal tumor invading the rectosigmoid region seems to be remarkably rare, as suggested by the report where the combined resection of the sigmoid colon and rectum had not been performed at all among the 42 cases of appendiceal carcinoma with multivisceral resection due to locally advanced tumor growth [30].

## 4. Conclusion

Although an appendiceal tumor invading the adjacent sigmoid colon or rectum is a rare clinical condition, a preoperative diagnosis can be obtained by colonoscopy and a CT scan with this condition in mind. A proper diagnosis facilitates the planning of a suitable surgical procedure, that is, en bloc resection of the ileocecal part and the rectosigmoid part.

## Figures and Tables

**Table 1:** Summary of reported cases and our case of appendiceal tumors invading the sigmoid colon or rectum.

Year	Author	Age/sex	Chief complaint	Invaded organ	Colonoscopic findings at SC or rectum	Biopsy at SC or rectum	CT findings	Preoperative diagnosis	Surgical procedure/postoperative therapy	Histological type WHO 2010	pTNM UICC 8th	Follow-up
1975	Andersson et al. [4]	82/F	Constipation, abdominal pain	SC	n.m.	n.m.	n.m.	n.m.	Ap, Sg	ANOS	n.m.	Dead (180 days)
1985	Yamada et al. [5]^#^	67/F	Abdominal mass	SC, ileum	n.m.	n.m.	n.m.	Cystomyxoma	ICR, closure of fistula/n.m.	AMN or MA	n.m.	n.m.
1990	Corder et al. [6]	67 /M	Hematochezia, syncope	SC	Devoid of mucosa	Granulation tissue	n.m.	n.m.	Ap, Sg, PC (en bloc)/no therapy	Low-grade AMN	n.m.	n.m.
1993	Kato et al. [7]^#^	48/M	Ileus symptoms	SC, bladder	Fistula	n.m.	A distended appendix filled with high-density substance	Appendiceal cancer	ICR, Sg, PC (en bloc)/AC	MA	T4bN1-2M1b (PMP)	Alive (n.m.)
1997	Ito et al. [8]^#^	49/M	Ileus symptoms	SC, ileum	Submucosal-tumor like lesion	MA	A mass connected with the vermiform appendix medial to the ileocecal part	Appendiceal cancer	ICR, HAR (en bloc)/AC	AMN	T4b N0 M0	Alive (1080 days)
1999	Tanakaya et al. [9]^#^	68/F	Hematochezia, abdominal pain	SC	Type 1 tumor (polypoid type)	n.m.	n.m.	SC cancer	Tumorectomy, colostomy/no therapy	AMN or MA	T4b NX M1b (PMP)	Dead (33 days)
2004	Sano et al. [10]^#^	89/F	Hematochezia	RS	Type 2 tumor (ulcerated type with clear margin)	ANOS	No findings of metastasis	Rectal cancer	ICR, LAR, etc. (en bloc)/no therapy	AMN or MA, partly ANOS	T4b N0 M0	Alive (n.m.)
2007	Kumon et al. [11]^#^	80/F	Hematochezia	RS	Rough and red mucosa, stenosis	ANOS	A rectal mass with heterogeneous enhancement continuous with appendix	Rectal cancer	ICR, AR (en bloc)/no therapy	AMN or MA	T4b N0 M1c (no. 251)	Alive (150 days)
2009	Murphy and Matar [12]	38/M	Hematochezia, abdominal pain	SC, ileum, bladder	n.m.	n.m.	A complex mass, arising in the lower abdomen, incorporating the caecum, small intestine, and sigmoid colon	Crohn's disease, SC, or ileal cancer	RHC, Sg, PC (en bloc)/AC	“Moderately differentiated, partly MA”	T4b N0 M0	Alive (300 days)
2009	Stojanovic et al. [13]	55/F	Hematochezia	SC	External compression, tumor infiltration	n.m.	Sigmoid colon infiltrated by 6 cm × 6 cm hypodense irregular tumor mass connected to the end of the appendix	Appendicular cancer	Laparoscopic RHC, Sg (en bloc)/no therapy	MA	T4b N0 M0	Alive (60 days)
2009	Mori et al. [14]^#^	58/F	Abdominal discomfort	SC	Elevated lesion	Granulation tissue	A 1 cm width dilated appendix	Appendiceal cancer	RHC, Sg/AC	AMN or MA	T4b N0 M0	Alive (600 days)
2013	Shibata et al. [15]^#^	44/M	Hematochezia, diarrhea	SC	Stenosis	ANOS or MA	A pelvic tumor extended from the ileocecal part to the sigmoid colon	SC cancer or appendiceal cancer	Bypass, sigmoidostomy/chemotherapy	“Atypical, mucin-producing adenocarcinoma”	T4b NX M1b	Dead (210 days)
2016	Fitzgerald et al. [16]	75/F	Hematochezia	Rectum	Fingerlike/frond-like polyp	Adenoma	A dilated appendix with its tip near the rectum	n.m.	Ap, AR (en bloc)/n.m.	“Adenocarcinoma”	T4b N0 M0	n.m.
2016	Hakim et al. [17]	68/M	Abdominal pain, constipation	SC, cecum	n.m.	n.m.	A dilated appendix with an 8 cm long, ovoid, periappendiceal mass containing a fistula to sigmoid colon	n.m.	Cecectomy, Ap, Sg, PC etc. (en bloc)/no therapy	MA	T4b N0 M0	Alive (360 days)
2018	Takahashi et al. [18]	13/F	Hematochezia, abdominal pain	SC, uterus, ovary	n.m.	n.m.	A mass with fecalith in the pelvic and right lower abdominal cavity	Acute appendicitis	ICR, Sg/AC	“Primary appendiceal adenocarcinoma”	T4b N0 M0	Alive (1800 days)
2018	Tokai et al. [19]^#^	66/M	Positive fecal occult blood test	SC	Elevated lesion	MA	A 7 x 4 cm sized cystic tumor adjacent to the sigmoid colon and cecum	Appendiceal cancer	ICR, Sg, ileectomy (en bloc)/AC	MA	T4b N1 M0	Alive (900 days)
	Our case	69/F	Hematochezia, anorexia	SC	Type 1 tumor (polypoid type)	ANOS	An appendiceal mass at the distal part of appendix, involving sigmoid colon	Appendiceal cancer	ICR, Sg (en bloc)/no therapy	ANOS	T4b N0 M0	Alive (600 days)

AC: adjuvant chemotherapy; ANOS: adenocarcinoma not otherwise specified; AR: anterior resection; Ap: appendectomy; F: female; ICR: ileocecal resection; AMN: appendiceal mucinous neoplasm; M: male; MA: mucinous adenocarcinoma; n.m.: not mentioned; PC: partial cystectomy; PMP: pseudomyxoma peritonei; RHC: right hemicolectomy; RS: rectosigmoid junction; SC: sigmoid colon; Sg: sigmoidectomy; #: written in Japanese.

## Data Availability

Data sharing is not applicable to this article as no datasets were generated or analyzed during the current study.
